# Bidialectalism and Bilingualism: Exploring the Role of Language Similarity as a Link Between Linguistic Ability and Executive Control

**DOI:** 10.3389/fpsyg.2018.01997

**Published:** 2018-10-23

**Authors:** Jessica Oschwald, Alisa Schättin, Claudia C. von Bastian, Alessandra S. Souza

**Affiliations:** ^1^University Research Priority Program “Dynamics of Healthy Aging”, University of Zurich, Zurich, Switzerland; ^2^Cognitive Psychology Unit, Department of Psychology, University of Zurich, Zurich, Switzerland; ^3^Department of Psychology, University of Sheffield, Sheffield, United Kingdom

**Keywords:** bidialectalism, bilingualism, language similarity, executive functions, linguistic processing

## Abstract

The notion of bilingual advantages in executive functions (EF) is based on the assumption that the demands posed by cross-language interference serve as EF training. These training effects should be more pronounced the more cross-language interference bilinguals have to overcome when managing their two languages. In the present study, we investigated the proposed link between linguistic and EF performance using the similarity between the two languages spoken since childhood as a proxy for different levels of cross-language interference. We assessed the effect of linearly increasing language dissimilarity on linguistic and EF performance in multiple tasks in four groups of young adults (aged 18–33): German monolinguals (*n* = 24), bidialectals (*n* = 25; German and Swiss German dialect), bilinguals speaking two languages of the same Indo-European ancestry (*n* = 24; e.g., German-English), or bilinguals speaking two languages of different ancestry (*n* = 24; e.g., German-Turkish). Bayesian linear-mixed effects modeling revealed substantial evidence for a linear effect of language similarity on linguistic accuracy, with better performance for participants with more similar languages and monolinguals. However, we did not obtain evidence for the presence of a similarity effect on EF performance. Furthermore, language experience did not modulate EF performance, even when testing the effect of continuous indicators of bilingualism (e.g., age of acquisition, proficiency, daily foreign language usage). These findings question the theoretical assumption that life-long experience in managing cross-language interference serves as EF training.

## Introduction

Bilingualism, or the active use of two languages from an early age on, has been suggested to have both positive effects on non-linguistic and negative effects on linguistic processing (Bialystok et al., 2012). On the one hand, the increased attentional demand bilinguals face when they have to select words in the appropriate language while inhibiting their second language (L2) is assumed to serve as lifelong training of executive control (Kroll and Bialystok, 2013) leading to better executive functions (EF) in bilinguals as compared to monolinguals. Bilingual advantages have been reported for several aspects of EF (Miyake et al., 2000): *inhibition* of prepotent responses (Bialystok et al., 2008; Salvatierra and Rosselli, 2010), *shifting* between mental sets and tasks (Prior and MacWhinney, 2010; Wiseheart et al., 2016), and *updating* and monitoring of working memory (WM) contents (Luo et al., 2013; Blom et al., 2014). On the other hand, the need to maintain more than one lexicon is assumed to come with disadvantages in lexical access (e.g., Bialystok, 2009), leading to worse linguistic performance in bilinguals as compared to monolinguals. Accordingly, it has been found that bilingual children and adults have a smaller receptive vocabulary (Bialystok et al., 2010; Bialystok and Luk, 2012), have lower scores in picture naming tasks (Gollan et al., 2005; Ivanova and Costa, 2008), and perform worse in word-fluency tasks (Gollan et al., 2002; Portocarrero et al., 2007) than monolinguals.

Whereas bilingual linguistic disadvantages are well-supported in the literature, bilingual EF advantages have been challenged by several recent replication failures (Morton and Harper, 2007; Paap and Greenberg, 2013; Antón et al., 2014; Duñabeitia et al., 2014; Gathercole et al., 2014; Kirk et al., 2014; Kousaie et al., 2014; Paap and Liu, 2014; de Bruin et al., 2015; Paap et al., 2016, 2014; von Bastian et al., 2016). This has led to a discussion of the variables potentially modulating the observation of bilingual EF advantages (Kroll and Bialystok, 2013; Baum and Titone, 2014; Valian, 2015). Recently, researchers have paid increasingly more attention to the multifaceted aspects of the bilingual experience, such as the age of L2 acquisition, language proficiency, and frequency of language use. Although these variables have been shown to modulate the performance of bilinguals in linguistic tasks (Portocarrero et al., 2007; Gollan et al., 2008; Luo et al., 2010; Blumenfeld et al., 2016), the importance of these factors in explaining bilingual EF advantages is still under debate. Two large-scale studies failed to observe a relation between age of L2 acquisition, language proficiency, language usage, and number of learned languages in multiple indicators of EF (Paap, 2014; von Bastian et al., 2016), and other studies did not observe a relation between age of acquisition and inhibitory control (Linck et al., 2008; Pelham and Abrams, 2014). A couple of studies have, however, reported effects of balance of language usage on inhibitory control and on shifting (Woumans et al., 2015; Yow and Li, 2015; Verreyt et al., 2016), and of L2 proficiency on conflict monitoring (Singh and Mishra, 2013). A further aspect of the bilingual experience that has received less attention in the literature is the similarity of the two languages spoken.

### The Role of Language Similarity

Evidence from event-related potential studies suggests that, in bilinguals, both languages are constantly activated even if only one of them is currently in use (Kroll et al., 2012). An explanation for this parallel activation is proposed by the BIA+ model, which suggests that bilinguals have a shared mental lexicon for both languages. Consequently, when recognizing a word, lexical representations that share orthographic, phonologic and/or semantic similarity with the target word are automatically activated regardless of the language they correspond to (Dijkstra and van Heuven, 2002). This non-selective activation is assumed to demand general executive control mechanisms to manage cross-linguistic activation (Coderre and van Heuven, 2014). Furthermore, this parallel activation leads to bidirectional cross-language interactions, such that the first language (L1) adapts to the grammar and words of L2, and vice versa (Kroll et al., 2014). Importantly, empirical evidence has shown that cross-language interactions vary as a function of overlap during word production (Schwartz et al., 2007) and reading (Van Assche et al., 2011). If cross-language interactions vary with the similarity of the two languages spoken, language similarity may have a profound impact on how much executive control is required to effectively use L1 and L2. Basically, language similarity could affect executive control demands in language selection in two ways. First, similar L1 and L2 could lead to stronger cross-language interference. If so, selecting the appropriate language should become more difficult the more similar the two languages are, thereby requiring more executive control to inhibit the interfering language, to reduce the costs of switching between languages, and to monitor the contents that get access to WM (Linck et al., 2008; Barac and Bialystok, 2012; Coderre and van Heuven, 2014). In this case, bilinguals with similar languages would train to exert executive control more intensively, leading to enhanced performance in EF tasks. Alternatively, it may be that similar languages yield more adaptation between languages, thereby facilitating lexical access and language comprehension due to their shared grammar, syntax, and phonology. If so, speaking two highly similar languages should reduce the need to exert executive control compared to speaking two more dissimilar languages. In this latter scenario, dissimilar languages would require stronger attentional control, increase the cost of switching between languages, and demand more monitoring of WM contents, thereby yielding more training of EF. In this case, bilinguals speaking similar languages would be less advanced in EF than those speaking dissimilar languages. These opposing views can be disentangled by assessing the impact of language similarity on both linguistic and EF tasks. By taking linguistic performance as a measure of the degree to which the two languages interfere with each other and, hence, of how much executive control is required for managing L1 and L2, it is possible to predict how language similarity modulates bilingual EF advantages.

There is evidence of facilitating effects of language similarity in bilingual children (Bialystok et al., 2003, 2005; Barac and Bialystok, 2012). However, a study with young adults has not found any differences in language switching costs as a function of language similarity (Costa et al., 2006). Thus, more evidence is needed to test for the effect of language similarity on linguistic performance in adulthood. Regarding EF performance, only a small number of studies assessed the impact of language similarity, with mixed results. Three studies found no effect of language similarity on EF performance: one study tested bilingual children on a shifting task (Barac and Bialystok, 2012); one study tested young adults (Linck et al., 2008) and another study tested older adults (Kirk et al., 2014) on an inhibition task. Yet another study with a sample of young adults yielded inconclusive results on an inhibition task (Coderre and van Heuven, 2014): bilinguals with dissimilar languages showed the smallest interference score in a Stroop task, but they also responded more slowly on the task. In an attempt to replicate this result, Paap et al. (2015) assessed Stroop performance in young adult monolinguals and three groups of bilinguals with varying script similarity. However, script similarity affected neither Stroop interference nor overall reaction times (RT). Instead, orthographic overlap between the two languages spoken was associated with slower RTs in the Stroop task (but not with Stroop interference). Taken together, the evidence for an effect of language similarity on EF performance is mixed.

An extreme form of language similarity is bidialectalism (i.e., speaking a dialect in addition to a standard language). Dialects are naturally tightly related to their originating languages, while still having a distinct grammar and phonology (Chambers and Trudgill, 1998). Yet, only few studies have related bidialectism to bilingualism. Antoniou et al. (2016) assessed performance in several EF tasks in children that were monolinguals, bidialectals, or bilinguals. Bilinguals and, to some extent, bidialectals outperformed monolinguals in a composite measure of WM and inhibitory control. Noteworthy, the EF advantage of bidialectals was weaker than that of the bilinguals, and only reached significance after covarying children’s verbal capacity. In contrast, Ross and Melinger (2017) tested monolingual, bidialectal, and bilingual children in tasks measuring inhibitory control and shifting. Bidialectalism did not yield a benefit in either measure, but bilinguals responded more accurately in one inhibition task. In a sample of older adults, Kirk et al. (2014) found that bidialectals performed similarly as monolinguals in a Simon task. To the best of our knowledge, there is no study focusing on young adulthood.

Taken together, the little research to date on the effects of language similarity on linguistic and EF tasks led to mixed results. The variability across studies may be due to several factors. First, most studies assessed either EF or linguistic performance, but not both (with exception of Barac and Bialystok, 2012). This makes it difficult to establish the link between language processing demands and executive control. Second, most studies assessed performance in only one task, and studies vary in terms of the ability assessed (e.g., inhibition, shifting). Recently, it has been proposed that bilingualism may have a subtle impact on diverse EF measures (Kroll and Bialystok, 2013). Hence, a broader assessment of EF might be required to uncover the effects of language similarity. Third, single-task assessments may also confound task-specific variance with ability-level effects (Shipstead et al., 2012). Thus, studies including multiple tasks measuring the same ability may provide more reliable and generalizable results.

### The Present Study

As reviewed above, there is little research on the effects of language similarity on EF and linguistic processing, particularly among young adults. In the present study, we investigated performance in these two domains simultaneously to examine whether and how language similarity mediates the relationship between language control and executive control. We hypothesize that there are two possible scenarios. Similar languages may lead to more linguistic interference and, thus, require increased executive control relative to dissimilar languages (Linck et al., 2008; Barac and Bialystok, 2012; Coderre and van Heuven, 2014). Alternatively, similar languages may interfere less with each other due to cross-linguistic adaptation. Adaptation should facilitate linguistic processing in more similar languages, thus requiring less executive control compared to dissimilar languages (Barac and Bialystok, 2012). Either way, both views imply that language similarity has opposite effects on EF and linguistic tasks: the conditions that lead to better linguistic performance should yield least training of EFs, thereby limiting EF benefits. The main goal of the present study was to provide a first assessment of this link by measuring how language similarity affects both linguistic and EF performance.

We assessed the effect of language similarity in EF and linguistic tasks by comparing performance of monolinguals, bidialectals, and bilinguals with language combinations that varied in the similarity to Standard German. As this was the first attempt to test the impact of language similarity on the link between EF and linguistic performance, no evidence was available as to what linguistic properties on what level (i.e., orthographical, phonological, semantic) would be most critical for language similarity to yield the hypothesized effects. Hence, we chose a more general measure of language overlap by categorizing languages as similar based on their language family. We considered languages within the Indo-European family as more closely related to each other than they are to languages of any other family based on some overlapping vocabulary and similarities in their general macro-structural syntax and grammar. In contrast, Indo-European and Non-Indo-European languages tend to differ in those aspects to a larger degree (Campbell, 2008; Comrie, 2008; Longobardi et al., 2013). Accordingly, our assumption was that individuals who speak languages from the Indo-European family would broadly deal with languages that share more linguistic properties than individuals whose languages stem from different families^1^. Several studies have shown bidirectional cross-linguistic interactions indicating that the multiple languages an individual speaks affect each other (Hohenstein et al., 2006; Brown and Gullberg, 2008, 2010, 2011; Ameel et al., 2009; Van Assche et al., 2011). Based on the BIA+ model, shared linguistic properties should lead to the activation of more lexical representations in the bilingual lexicon that show overlap with the target word, thus resulting in increased cross-linguistic interactions for more similar languages (Dijkstra and van Heuven, 2002). Hence, the broad similarity of the languages may facilitate or hinder language processing and in turn demand less or more EF. Taking the present definition of language similarity, we assume that Standard German and the Swiss-German dialect share the highest degree of overlap (e.g., shared vocabulary, phonology, syntax, etc.). In contrast, when one compares Standard German to other languages from the Indo-European family (e.g., German and English, or German and French), there are much less shared properties (e.g., vocabulary), but there remain some macro-structural similarities such as phonological and syntactic processes that could impact the learning and daily usage of these language combinations. Languages from different language families (e.g., German and Turkish, or German and Chinese), conversely, are assumed to share even less properties than languages within the same language family (e.g., different vocabulary, different phonology, etc.). We assumed that there should be less cross-linguistic interactions between these more dissimilar languages than between the (relatively) more similar languages within the Indo-European family.

Having these considerations in mind, participants were classified as belonging to one of four groups. The *monolingual* group comprised native speakers of Standard German only. The *bidialectal* group comprised native speakers of Standard German and the Swiss German dialect. The Swiss German dialect is very closely related to Standard German, as both belong to the German languages within the Indo-European language family, and are located on neighboring branches of the family tree (Simons and Fennig, 2018). The bidialectals in our group used both the Swiss German dialect and Standard German in most social contexts. Bilinguals were speakers of Standard German (and, in most cases, also of the Swiss German dialect) and learned another language. We included a group of bilinguals proficient in Standard German and another Indo-European language (hereafter *similar bilinguals*, e.g., English, French, or Italian), and a group of bilinguals proficient in Standard German and a Non-Indo-European language (*dissimilar bilinguals*, e.g., Arabic, Turkish, or Chinese). Performance of these four groups was compared in three tasks aimed at assessing their linguistic ability and in several measures of EF that have been linked to inhibitory control, monitoring, shifting, mixing, and WM (von Bastian et al., 2016).

In sum, our four language groups differ progressively in terms of which additional languages they spoke. Monolinguals spoke only Standard German, bidialectals spoke Standard German and the Swiss German dialect, and bilinguals spoke Standard German, the Swiss German dialect, and another language that was of the same Indo-European family (similar bilinguals) or not (dissimilar bilinguals). Hence, this partition assumes that speaking additional languages has an additive effect with speaking the dialect. This might not be the case, and it is also conceivable that speaking the dialect and speaking an additional language have opposite effects that cancel each other, thereby diluting group differences. This was a risk of our design. However, this would be an actual concern only if we would find no effects of language similarity neither in linguistic nor in EF performance, or if the effect was constrained only to the comparison between monolinguals and bidialectals with no further differences for the bilingual groups. To foreshadow our results, we did obtain a monotonic effect of language similarity on linguistic performance, which is in line with the assumption that speaking the dialect and an additional language have additive effects.

## Materials and Methods

Participants signed up for the study via an online form determining their eligibility for study participation (physically and psychologically healthy, not color-blind, and speaker of Standard German). Eligible participants were invited via e-mail to complete an online language history questionnaire (completed in Standard German). Next, they were invited for a laboratory session where they completed a battery of tasks taking approximately 2 h, with a 10-min break midway. All tasks were presented in Standard German. During the laboratory session, participants first completed the Ishihara test for color blindness (Ishihara, 2003), followed by two paper-pencil tasks measuring linguistic ability (a word completion test and a verbal fluency test). Then, they were asked to complete a computerized test battery comprising 11 cognitive tasks. The test battery was programmed with Tatool, an open-source software for programming psychological experiments (von Bastian et al., 2013). To avoid order and fatigue effects, half of the participants completed the computer-based tasks in reversed order (von Bastian and Oberauer, 2013). Participants were randomly assigned to the task order, equally balanced across language groups.

### Participants

One-hundred and eleven young adults voluntarily took part in the study. Participation was compensated with extra-course credit or 40 CHF (about 40 USD). Participants were students at a Swiss university or held a diploma comparable to a Swiss high-school certificate (Matura). Written informed consent was obtained for all participants. Participants were tested in groups of up to five. The experimental protocol was approved by the Ethics Committee of the Faculty of Arts and Social Sciences of the University of Zurich (in accordance with the Helsinki declaration), and participants were debriefed at the end of the study. Twelve participants were excluded from the analysis for various reasons: (a) they were not proficient in German (*n* = 1), (b) reported language combinations that did not match the pre-defined language groups (*n* = 7)^2^, or (c) did not fulfill the requirements of our definition of bilingualism described below (*n* = 4). Thus, the final sample consisted of 99 participants, aged 18–33 years (M = 23.5, *SD* = 3.69). The sample characteristics are listed in Table 1.

**Table 1:** Demographic characteristics by language group.

Measure	Monolinguals *n* = 25		Bidialectals *n* = 26		Similar bilinguals *n* = 24		Dissimilar bilinguals *n* = 24		Evidence for/against group differences	
					
	*M*	*SD*	*M*	*SD*	*M*	*SD*	*M*	*SD*	BF_10_	% error
Gender (f/m)	20/5		21/5		16/8		19/5		**0.15**	±0.00
Age	24.48	3.55	23.46	4.32	22.50	3.24	23.38	3.47	**0.20**	±0.00
Education (1–4)^a^	2.08	0.95	1.69	0.97	1.54	0.88	1.63	0.92	**0.32**	±0.00
Parents’ education (1–8)^b^	5.54	1.42	4.42	1.47	5.02	1.58	4.19	2.10	2.07	±0.00
Migration background (*n*)^c^	22		2		8		10		**>1,000**	±0.00
RAPM	0.63	0.21	0.70	0.16	0.58	0.29	0.61	0.27	**0.19**	±0.00


*RAPM, Raven’s Advanced Progressive Matrices. Values printed in bold indicate at least substantial evidence for (BF_*10*_ ≥ 3) or against (BF_*10*_ ≤ 0.33) group differences.*

*^*a*^Highest completed degree [1, matura degree (higher education entrance qualification); 2, university of applied sciences degree; 3, university degree; 4. doctoral degree].*

*^*b*^Average of mother’s and father’s educational degree (1, no or less than 9 years of school; 2, mandatory school of approx. 9 years; 3, vocational education; 4, further professional education; 5, matura degree; 6, university of applied sciences; 7, university degree; 8, doctoral degree).*

*^*c*^Country of residence differs from country of origin.*

**FIGURE 1:**
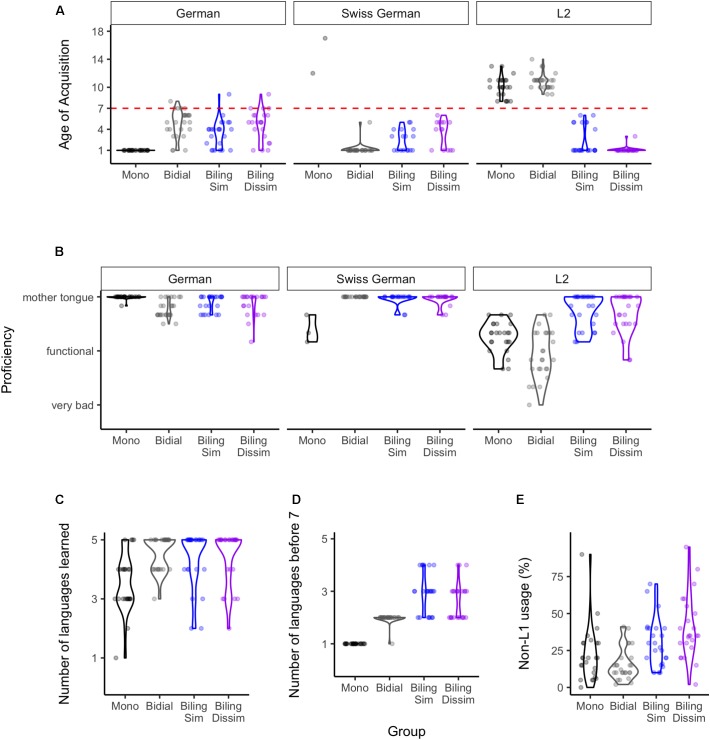
Distribution of the self-reported language variables in each group. **(A,B)** show age of acquisition and proficiency, respectively, in German, Swiss German, and the L2. **(C)** shows the total number of languages learned, whereas **(D)** shows the number of languages learned before the age of seven. Finally, **(E)** presents the daily percentage usage of languages other than German/Swiss German.

Participants were classified into one of four language groups. *Monolinguals* (*n* = 25) were native speakers of only Standard German and had limited knowledge of the Swiss German dialect. *Bidialectals* (*n* = 26) were native speakers of the Swiss German dialect and Standard German. In the German speaking part of Switzerland, the Swiss German dialect is used in most daily interactions and is typically the language children will learn first at home. However, Standard German is used when interacting with non-dialect speakers, in the news, on most of the available TV channels, and in some other social contexts. Thus, Swiss Germans are highly proficient in both the Swiss German dialect and Standard German. Moreover, the Swiss German dialect is a spoken dialect only, with Standard German being the written language that is obligatory in formal contexts. Hence, all children must learn Standard German when entering school at the age of 6 or 7 years. Participants in the monolingual and bidialectal groups had also formal foreign language education during secondary school (most commonly English or French starting on average after the age of 10), but achieved much lower proficiency in these languages (see Figure 1). The two remaining groups were bilinguals. Participants qualified as bilinguals if they (1) learned at least one language (henceforth L2) in addition to Standard German and/or Swiss German up to the age of seven (i.e., before entering school and any formal foreign language education), and (2) indicated that they were still actively using their L2. This definition of bilingualism is in line with the inclusion criteria used in several prior studies (e.g., see Bialystok et al., 2006; Costa et al., 2009; Hernández et al., 2010; Luk et al., 2011; Gold et al., 2013). In sum, what separates our bilingual participants from their monolingual and bidialectal counterparts is the early onset of bilingualism, and also the greater proficiency they achieved in their languages. Bilinguals with an L2 from the Indo-European language family were classified as *similar bilinguals* (*n* = 24), and bilinguals with an L2 from a Non-Indo-European language family were classified as *dissimilar bilinguals* (*n* = 24). Similar bilinguals were native speakers of English (7), French (3), Italian (3), Polish (2), Portuguese (2), Spanish (3), Rhaeto-Romanic (3), or Albanian (1). Dissimilar bilinguals were native speakers of Chinese (2), Korean (1), Laotian (1), Tagalog (1), Tamil (1), Tibetan (1), Turkish (7), Hungarian (6), Finnish (1), Arabic (2), or Malayalam (1).

The language groups were matched in terms of gender, age, educational background, and Raven’s Advanced Progressive Matrices (RAPM) scores as confirmed by substantial evidence against group differences in univariate Bayesian ANOVAs (see Table 1). The evidence regarding group differences in socioeconomic status (SES) was ambiguous. Paired contrasts between groups revealed, however, that all groups were comparable in terms of their SES, except for the parents of monolinguals having, on average, higher educational degrees than parents of bidialectals. Groups differed though regarding their migration background: Most monolinguals and more than a third of the bilinguals, but less than 8% of the bidialectals were currently residing in a different country than their country of origin.

### Language and Demographic Assessment

Demographic and language background information were assessed with a questionnaire administered online using SoSci Survey (Leiner, 2018). The questionnaire was based on the language history questionnaire from Li et al. (2006), translated to German, and adapted for the purposes of this study by two of the authors (JO and AS). After assessing demographic variables and SES, participants were asked to report all languages they have learned (up to a maximum of five languages) in the order in which they had learned them, starting with their native language. Participants were explicitly instructed to consider the Swiss German dialect as a separate language. In addition, they were asked to indicate detailed information on the usage of each language. For the present purposes, we extracted the self-reported age of acquisition, proficiency, and percentage of daily language usage for German, Swiss German dialect, and each participant’s L2 (language other than German and/or the Swiss German dialect acquired earliest) from the questionnaire. Previous research obtained high correlations of self-rated proficiency measures with objective assessments of language proficiency (Luk and Bialystok, 2013). Accordingly, we used the above listed self-reported measures to describe the language experience of our groups, and also as continuous predictors of performance in our tasks.

Figure 1 presents the distribution of language background variables in each language group. Panel A indicates the self-reported age of acquisition of each language of interest here, namely German, Swiss German dialect, and the L2. There are clear differences between the language groups, particularly with regards to age of L2 acquisition, which was the inclusion criterion for bilinguals in this study. Note that with regards to the Swiss German dialect, only two monolinguals reported having learned the dialect. Complementarily, panel B presents self-reported proficiency in each of these languages. Again, language groups differed substantially particularly with regards to L2 proficiency: bilinguals reported higher proficiency than monolinguals or bidialectals. The two monolinguals that reported learning Swiss German also reported that their proficiency on the dialect was lower than that of a native speaker. Panel C shows that most participants in the study learned more than one language at some point in their lives (note that Swiss German is included here as an additional language). Panel D indicates, however, that the age of acquisition of the learned languages differed between groups: monolinguals acquired only one language by the age of 7, whereas bidialectals acquired two languages (i.e., the Swiss German dialect and Standard German), and bilinguals (similar and dissimilar groups) acquired two or more languages (i.e., the Swiss German dialect and/or Standard German and the L2). Lastly, panel E shows that participants in all groups reported using a foreign language (i.e., another language besides the Swiss German dialect or Standard German) for a substantial part of their day. This is probably the case because all participants were university students, and they were confronted with English on a daily basis. Importantly, the item did not differentiate between active (e.g., speaking) and passive (e.g., listening) non-L1 usage. In sum, our bilinguals learned more languages at an earlier age and with higher proficiency than monolinguals or bidialectals.

### Linguistic and EF Assessment

Linguistic ability was assessed with three tasks, and the five EF abilities (inhibition, monitoring, mixing, shifting, and WM) each with two tasks using different materials to reduce the influence of task-specific variance. Furthermore, we included a short non-verbal reasoning test to assess group comparability on this ability. All tasks were preceded by practice trials which were excluded from the final analysis. Dependent measures were coded so that larger values indicate better performance.

### Linguistic Ability

**FIGURE 2:**
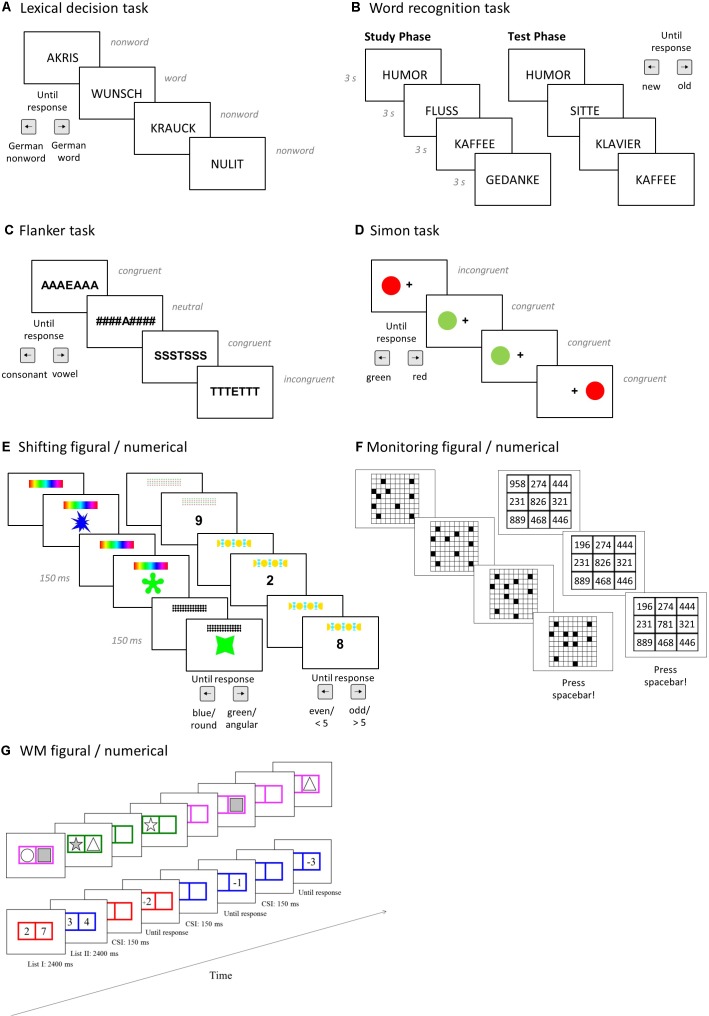
Graphical representation of the tasks administered. See text for details. CSI, cue-stimulus interval. **(A,B)** Linguistic tasks. **(C,D)** Inhibition tasks. **(E)** The figural and numerical versions of the shifting tasks. **(F)** The figural and numerical versions of the monitoring tasks. **(G)** The figural and numerical versions of the working memory (WM) tasks.

Bilinguals have been consistently found to be disadvantaged in linguistic tasks compared to monolinguals: they produce less words in semantic fluency tasks (Gollan et al., 2002; Portocarrero et al., 2007) and react slower and less accurately in lexical decision tasks (Ransdell and Fischler, 1987; Lehtonen et al., 2012). Moreover, Ransdell and Fischler (1989) found that bilinguals benefitted less from accessing concrete in comparison to abstract words. Hence, we assessed linguistic ability through performance in the three tasks: verbal fluency, lexical decision-making, and the concreteness effect in a word recognition task. All tasks were conducted in Standard German language. In the *verbal fluency task* (administered in paper-and-pencil format), participants were asked to write down as many German words as they could think of in response to a categorical prompt (i.e., animals, fruits, clothes, musical instruments, objects on wheels, and furniture) within 2 min for each category. Words from the same semantic subcategory (e.g., poodle and labrador from the subcategory dogs), or words with the same meaning (e.g., “Orange” and “Apfelsine” both of which refer to an orange in Standard German) were coded as one word only. Linguistic accuracy in the verbal fluency task was measured via the sum of unique words (average across two coders) generated across all semantic categories. Participants also completed a word-fragment completion test (Jäger et al., 1997) that was administered as a warm-up for the following verbal fluency test. Data of this task were discarded and not further analyzed. For the remaining two tasks, we derived two performance measures, namely the accuracy with which the task was completed (hereafter referred to as linguistic accuracy) and the speed of processing (linguistic speed). In th*e lexical decision task*, participants indicated with a key press whether a visually presented string was a word (right arrow key) or a non-word (left arrow key). The stimulus remained onscreen until a response was made (see Figure 2A). Participants completed 128 trials consisting of a pseudo-random sequence of 64 German words and 64 non-words, matched regarding their number of letters and syllables, and their frequency (only words) using a semantic atlas for German words (Schwibbe et al., 1981). We calculated linguistic speed using the mean log-transformed RTs (multiplied by -1, so that higher values represent better performance), and linguistic accuracy via detection performance computed as *d’* = *z*(H)-*z*(FA), with H being the hit rate, FA being the false alarm rate, and *z* reflecting the *z*-transformation of these values. In the *word recognition task*, participants were instructed to memorize 30 German nouns presented sequentially (3 s each) on the screen (see Figure 2B). Half of the nouns were concrete (e.g., elephant) and half of them abstract (e.g., theory). Subsequently, participants were sequentially shown 60 probe words, including the 30 previously presented words (old) and 30 new words (new), randomly intermixed. Participants decided whether the probe word was old (right arrow key) or new (left arrow key). Each probe word remained onscreen until a response was made. The concreteness benefit (i.e., the performance difference in responding to abstract and concrete words) in log-transformed RTs was used as a measure of linguistic speed, and the concreteness benefit in detection performance (*d*’) was used as a measure of linguistic accuracy. Both measures were coded so that a larger value reflects a larger concreteness benefit.

### Inhibition

Bilinguals’ extensive practice inhibiting their currently irrelevant language (Green, 1998) is assumed to yield advantages in inhibiting irrelevant information in non-linguistic tasks. We used two tasks to assess inhibition. In the *flanker* task, participants indicated as fast and accurately as possible whether the central letter (target) in a string of seven letters was a vowel (left arrow key) or consonant (right arrow key). The stimulus remained onscreen until a response was given, followed by an inter-trial interval (ITI) of 250 ms (see Figure 2C). Participants completed 144 trials. In one third of the trials, the letters flanking the target were congruent (target and flankers require the same response, e.g., “SSSTSSS” or “EEEAEEE”), incongruent (target and flankers require the opposite response, e.g., “SSSASSS” or “EEETEEE”), or neutral (flankers are irrelevant to the task, e.g., “###S###” or “###A###”). As an inhibition index, we computed the difference between the log-transformed RTs in neutral and incongruent trials. In the *Simon task*, each trial started with a fixation cross presented centrally for 250 ms, followed by a colored circle appearing on the left or right side of the screen (see Figure 2D). Participants had to indicate as fast and as accurately as possible whether the circle was red (right arrow key) or green (left arrow key). The circle remained onscreen until a response was made, followed by an ITI of 250 ms. Participants completed 200 trials: 75% were congruent, that is, the location of the response (e.g., left) and the spatial location of the stimulus (e.g., left) matched, and 25% were incongruent trials in which the spatial location of the response and of the stimulus did not match. As an inhibition index, we computed the difference between the log-transformed RTs in congruent and incongruent trials.

### Shifting and Mixing

Bilinguals are also assumed to benefit from the extensive practice in switching between languages that generalizes to shifting between tasks, yielding smaller non-linguistic task-switch costs (for a review see Yang et al., 2016). In addition, bilinguals are assumed to excel in monitoring which task to apply in which situation (Soveri et al., 2011; Wiseheart et al., 2016), which is reflected by mixing costs that can also be assessed with shifting tasks. Therefore, we used the figural and a numerical switching tasks from von Bastian et al. (2016) consisting of single-task blocks, where only one task is performed, and a mixed-task block in which two tasks switch unpredictably. In the *color-shape task*, participants classified bivalent figural stimuli according to their color (blue or green) or shape (round or angular) by pressing the left (for blue or round) or right arrow key (for green or angular). The task included 32 angular and 32 round shapes, with half of each colored in blue or green, respectively. In the *parity-magnitude task*, participants classified digits from 1 to 9 (excluding 5) according to their parity (even or odd) or magnitude (smaller or larger than 5) by pressing the left (for even or smaller than 5) or right arrow key (for odd or larger than 5). In both task versions, the upcoming task rule was indicated by an abstract cue (e.g., patterned bar) presented on the top of the screen. After a cue-stimulus interval (CSI) of 150 ms, a shape or digit (depending on the task version) appeared in the center of the screen until participant’s response (see Figure 2E). Participants completed two single-task blocks (one for each task) of 64 trials each, followed by a mixed-task block of 129 trials, and again the two single-task blocks (in reversed order). The mixed-task block contained 50% repeat trials (i.e., trials in which the task in the current and preceding trial was the same), and 50% switch trials (i.e., the tasks in the current and the preceding trial were different). The first trial in the mixed block was excluded from analysis, as it was neither a repeat nor a switch trial. To assess shifting ability, switching cost scores were calculated by subtracting the average RT in switch trials from the average RT in repeat trials (both from the mixed-task block and log-transformed). To assess mixing, mixing cost scores were computed by subtracting the average repeat trials RT in the mixed-task block from the average RT in the single-task block (both log-transformed).

### Monitoring

Monitoring was measured with tasks requiring participants to sustain attention to a stream of inputs to detect certain patterns or relations. Participants completed two tasks from von Bastian et al. (2016) in which they had to monitor independently changing objects, and react whenever a predefined relation between these objects occurred (Oberauer et al., 2003; von Bastian and Oberauer, 2013). In the *squares task*, a display of 20 dots in a 10 × 10 grid was shown and, every 2 s, two dots randomly changed their position. Participants had to press the space key whenever four dots formed a square. In the *digits task*, a 3 × 3 grid with three-digit numbers in each cell was presented and, every 2 s, the numbers in one cell changed. Participants were instructed to press the spacebar whenever the last digits of the numbers in a row, column, or diagonal were identical (see Figure 2F). Both task versions comprised 16 trials, each presenting 2 to 8 changes before the predefined relation between objects appeared. The monitoring score was *d’*.

### WM

WM is assumed to be tightly related to executive control (Engle, 2002), which makes it one candidate EF domain for bilingual benefits (Bialystok, 2017). WM was assessed with a figural and numerical version of the list-switching paradigm (Oberauer, 2005; Oberauer et al., 2013; Gade et al., 2017) in which participants had to retain two memory lists in WM for ongoing processing (see Figure 2G)^3^. In the *figural task*, participants memorized two lists distinguished by a pink or green frame. Each list consisted of a row with two colored boxes each containing a filled shape (selected from a pool of 20 shapes with no replacement). The lists were presented sequentially (for 2400 ms each) with a 250 ms inter-list blank interval. Next, 13 memory probes followed. For each probe, the relevant list was cued by the color of the row of boxes and, 150 ms thereafter, the probe appeared in one of the boxes. Participants indicated a match between the probe and the memory item in the same list position (left arrow key, 50% of the trials) or a mismatch (right arrow key, 50% of the trials). Mismatch probes were shapes presented in another list or list position (50%), or not presented in the current trial at all (50%). The *numerical task* followed a similar task structure: participants memorized a red and a blue list, each consisting of two digits (ranging from 1 to 9). Again, a series of 13 memory probes followed in which the relevant list was cued by color, and 150 ms later an arithmetic operation was shown in one of the boxes (e.g., +2). Participants had to retrieve the item shown in this position of the relevant list, apply the arithmetic operation to it, and enter the result (which was always between 1 and 9) using the keyboard. Participants entered the results of the operation, but they were asked to remember the original value of the item. In both task versions, each sequence of memory list encoding followed by 13 probes was considered a run. Participants completed 12 runs, each containing 50% list-repeat trials (same list was tested in the current and the previous trial) and 50% list-switch trials (current and previous list were different). The WM scores were proportion of correct responses in both the figural and numerical version.

### Reasoning

To evaluate whether the language groups matched regarding their non-linguistic fluid intelligence, we administered a short (Arthur and Day, 1994) computerized version of Raven’s Advanced Progressive Matrices (Raven, 1990). Participants had 15 min to complete 12 patterns. For each pattern, they had to choose 1 out of 8 response alternatives. The number of correctly solved items (out of 12) served as dependent measure.

## Statistical Analysis

### Data Preprocessing

For RT based scores, we removed RTs associated with incorrect responses. Next, RTs were trimmed by removing outliers. Outliers were defined as RTs being three median absolute deviations away from the overall median (Leys et al., 2013). RTs were log-transformed to better approach normality before computing the relevant RT-based scores. To eliminate the unwanted source of variance introduced by having administered two test orders, we arbitrarily chose one order as the reference, and corrected the data of the other order for the mean difference between them (von Bastian and Oberauer, 2013; von Bastian et al., 2016). Lastly, all task scores were *z*-transformed.

### Bayesian Linear Mixed-Effects Modeling

We analyzed our data with Bayesian linear mixed-effects models. The advantage of using Bayesian statistics is that the evidence supporting both the alternative and the null hypothesis can be assessed. We used the BayesFactor package (Morey and Rouder, 2015) implemented in R (R Core Team, 2017), with the default prior settings (i.e., *r* = 0.50). The *lmBF* function implemented in the package computes the strength of the evidence for a specified model (M_1_) against a Null model (M_0_). For example, M_1_ may state that performance of monolinguals differs from bidialectals (alternative hypothesis), whereas M_0_ states that there is no group effect (null hypothesis). The ratio of the likelihood of these two models given the data is the Bayes factor (BF). The BF is the factor by which prior beliefs should be updated in light of the data. For example, a BF for M_1_ over M_0_ (hereafter, BF_10_) of 5 translates into the data being five times more likely under the alternative than under the null hypothesis. Likewise, BF_10_ = 0.2 means that the data are 5 times more likely under null hypothesis than the alternative hypothesis. When BF_10_ = 1, the data are equally likely under both hypotheses and, hence, the evidence is ambiguous. It is common to consider BF_10_ ≥ 3 as providing substantial evidence for the alternative hypothesis over the null, and BF_10_ ≤ 0.33 as providing substantial evidence for the null over the alternative hypothesis (Wagenmakers et al., 2011).

We tested for an effect of language similarity in each cognitive ability separately, using two approaches. First, we coded language similarity with a linear contrast over language group (using the *poly* function in R) and entered this variable as a fixed predictor in the models. This contrast implements the assumption that language groups differ in a monotonically decreasing fashion regarding language similarity. Second, to faciliate comparability to previous studies on effects of language similarity, we compared adjacent levels of language similarity (aka. sliding contrast; i.e., monolinguals vs. bidialectals, bidialectals vs. similar bilinguals, and similar bilinguals vs. dissimilar bilinguals). In addition, to test for bilingual effects more commonly investigated in the literature, we also contrasted the monolingual group with the similar and dissimilar groups (simple contrasts). For each model, we included random intercepts for participant and for task. We also included parents’ education level as a proxy for SES as a continuous predictor in all analyses (von Bastian et al., 2016). Two participants failed to provide information regarding their parents’ education level (one monolingual and one similar bilingual). To keep these participants in the sample, we replaced their missing values with the average SES of their respective groups. Excluding these participants from the analyses altogether did not substantially change the pattern of results. All analyses were computed with a high number of iterations (i.e., 400,000) to ensure that the error in estimating the BF was below 5%.

The data and analysis scripts for performing the analyses reported here are available at the Open Science Framework (OSF) at https://osf.io/uf2hs. The computer-based tasks used here (except the Raven’s) are freely and publicly available on Tatool Web (www.tatool-web.com). Supplementary Materials are available at the journal website (URL) and also at the OSF.

## Results

Descriptive statistics for all (non-transformed) measures as a function of language group are listed in Table 2. Zero-order correlations between measures and reliabilities are listed in Table 3. Split-half reliabilities (for difference scores, *d*’ and RTs; corrected with the Spearman-Brown prophecy formula) and Cronbach’s alpha (for accuracies) were within the acceptable range for all scores, except for the accuracy and speed scores derived from the word recognition task and the flanker inhibition score. All measures assessing the same ability were significantly positively intercorrelated, except for the linguistic accuracy scores (although the correlation between the verbal fluency and lexical decision task was marginally significant: *p* = 0.051, *r* = 0.20), linguistic speed scores (for which the correlation was negative), and the flanker and Simon inhibition scores. The evidence for all expected effects (concreteness effects, inhibition, mixing, and shifting costs) was substantial (see Table 4). To rule out that problems with reliability or lack of correlations between tasks were masking the effects of interest, we additionally ran all analyses on the level of individual tasks (see Supplementary Table S1).

**Table 2:** Descriptive statistics for each task score by language group.

Measure	Monolinguals		Bidialectals		Similar bilinguals		Dissimilar bilinguals	
				
	*M*	*SD*	*M*	*SD*	*M*	*SD*	*M*	*SD*
Linguistic accuracy								
Lexical decision	4.18	0.51	4.07	0.45	3.92	0.56	3.62	0.66
Word recognition	0.36	0.79	0.70	0.72	0.41	0.87	0.11	0.63
Verbal fluency	108.28	23.26	110.37	17.90	107.35	20.33	99.40	17.02
Linguistic speed								
Lexical decision	-6.56	0.14	-6.58	0.13	-6.57	0.18	-6.55	0.13
Word recognition	0.04	0.06	0.04	0.06	0.01	0.05	0.01	0.05
Inhibition								
Flanker	0.00	0.06	-0.02	0.04	-0.01	0.04	-0.01	0.04
Simon	-0.20	0.06	-0.18	0.08	-0.16	0.05	-0.17	0.06
Monitoring								
Figural	2.43	0.51	2.53	0.40	2.62	0.53	2.45	0.43
Numerical	2.25	0.55	2.58	0.66	2.61	0.85	2.66	0.82
Mixing								
Figural	-0.60	0.16	-0.51	0.21	-0.48	0.17	-0.55	0.22
Numerical	-0.31	0.19	-0.28	0.21	-0.26	0.16	-0.25	0.20
Shifting								
Figural	-0.33	0.22	-0.28	0.17	-0.37	0.19	-0.27	0.17
Numerical	-0.35	0.20	-0.36	0.19	-0.37	0.16	-0.33	0.17
Working memory								
Figural	0.78	0.08	0.83	0.08	0.82	0.09	0.80	0.11
Numerical	0.89	0.16	0.95	0.11	0.94	0.08	0.96	0.06


*See text for dependent measures of the variables listed.*

**Table 3:** Correlations between measures and reliabilities.

Variable	1	2	3	4	5	6	7	8	9	10	11	12	13	14	15
Linguistic accuracy															
1. Lexical decision	0.67														
2. Word recognition	0.10	-0.13													
3. Verbal fluency	0.20	0.09	0.80												
Linguistic speed															
4. Lexical decision	-0.11	-0.02	0.17	0.98											
5. Word recognition	0.11	0.11	0.12	-0.18	0.39										
Inhibition															
6. Flanker	-0.10	0.05	-0.07	-0.01	-0.01	0.40									
7. Simon	0.07	0.11	0.00	0.03	-**0.29**	0.00	0.81								
Monitoring															
8. Figural	**0.25**	0.02	**0.32**	0.00	-0.04	0.06	0.16	0.65							
9. Numerical	0.09	0.05	0.02	**0.26**	-0.17	-**0.34**	0.10	**0.22**	0.69						
Mixing															
10. Figural	-0.06	0.10	-0.12	-0.10	-0.11	0.13	0.08	0.07	0.13	0.96					
11. Numerical	-0.07	0.11	-0.07	0.06	0.08	0.00	-0.05	0.16	0.17	**0.48**	0.97				
Shifting															
12. Figural	0.01	-0.14	0.03	0.11	-0.03	-**0.21**	0.05	-0.14	0.01	-**0.64**	-**0.50**	0.90			
13. Numerical	0.04	0.02	-0.01	-0.09	-0.11	0.05	0.16	-0.09	0.02	-0.07	-**0.64**	**0.39**	0.93		
Working memory															
14. Figural	**0.37**	**0.21**	0.05	-0.04	-0.11	-0.08	0.05	**0.31**	**0.31**	**0.25**	**0.29**	-**0.24**	-0.19	0.81	
15. Numerical	0.08	-0.01	0.18	0.02	-0.05	-0.16	-0.06	0.16	**0.32**	**0.22**	**0.25**	-0.17	-0.15	**0.38**	0.89


*Correlation coefficients printed in bold were significant (*p* < 0.05). Reliabilities are printed along the diagonal.*

**Table 4:** Evidence for concreteness effects, inhibition, mixing and shifting costs.

Ability/Task	Trial type I		Trial type II		Difference (I-II)		Evidence	
				
	*M*	*SD*	*M*	*SD*	*M*	*SD*	BF_10_	% error
Linguistic	*abstract*		*concrete*		*concreteness*			
Word Rec. Acc	2.52	0.78	2.92	0.79	0.40	0.78	**>1,000**	±0.00
Word Rec. Speed	6.73	0.15	6.70	0.15	0.03	0.06	**752.90**	±0.00
Inhibition	*neutral/congruent*		*incongruent*		*inhibition costs*			
Flanker	6.29	0.12	6.31	0.11	-0.01	0.05	**4.29**	±0.00
Simon	6.07	0.12	6.24	0.12	-0.18	0.07	**>1,000**	±0.00
Mixing	*repetition (single)*		*repetition (mixed)*		*mixing costs*			
Figural	6.20	0.10	6.74	0.23	-0.54	0.20	**>1,000**	±0.00
Numerical	6.30	0.11	6.59	0.25	-0.28	0.19	**>1,000**	±0.00
Shifting	*repetition (mixed)*		*switch (mixed)*		*shifting costs*			
Figural	6.74	0.23	7.07	0.18	-0.31	0.19	**>1,000**	±0.00
Numerical	6.59	0.25	6.94	0.21	-0.35	0.18	**>1,000**	±0.00


*M, mean; *SD*, standard deviation; Word Rec., Word Recognition; Acc, accuracy. Values of trial type I and II are based on log-transformed reaction times (except for accuracies of the word recognition task), and are uncorrected for order-effects. The difference of trial type I and II reflects the concreteness effects, inhibition, mixing and switch costs, and is corrected for order-effects. Values printed in bold indicate at least substantial evidence for the alternative hypothesis (BF_*10*_ ≥ 3).*

**FIGURE 3:**
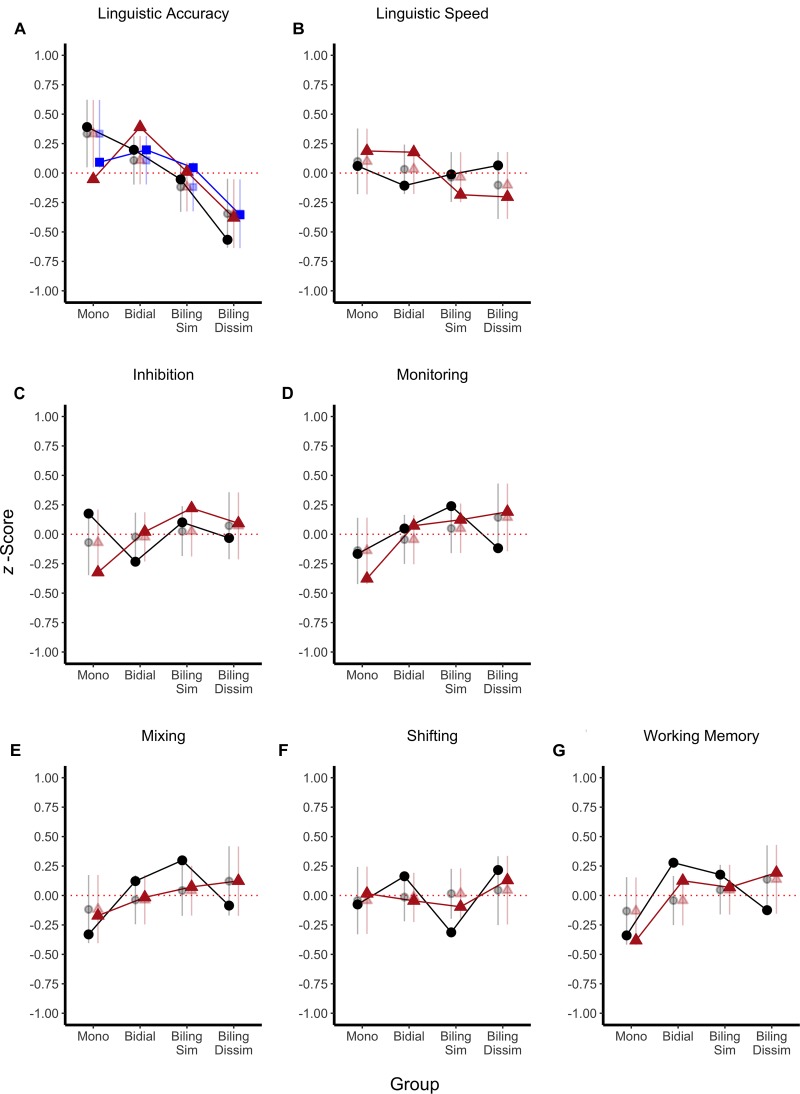
Task scores (*z*-transformed; solid dots) and model predictions (transparent dots and error bars) as a function of language group. Predictions are means (dot) and 95% HDI (error bars) of the posterior of the linear contrast over language similarity. Each panel **(A–G)** shows a different ability. Mono, monolinguals; Bidial, bidialectals; Biling, bilinguals; Sim, similar; Dissim, dissimilar; Lexic, lexical decision; Rec, recognition; Fluency, verbal fluency.

**FIGURE 4:**
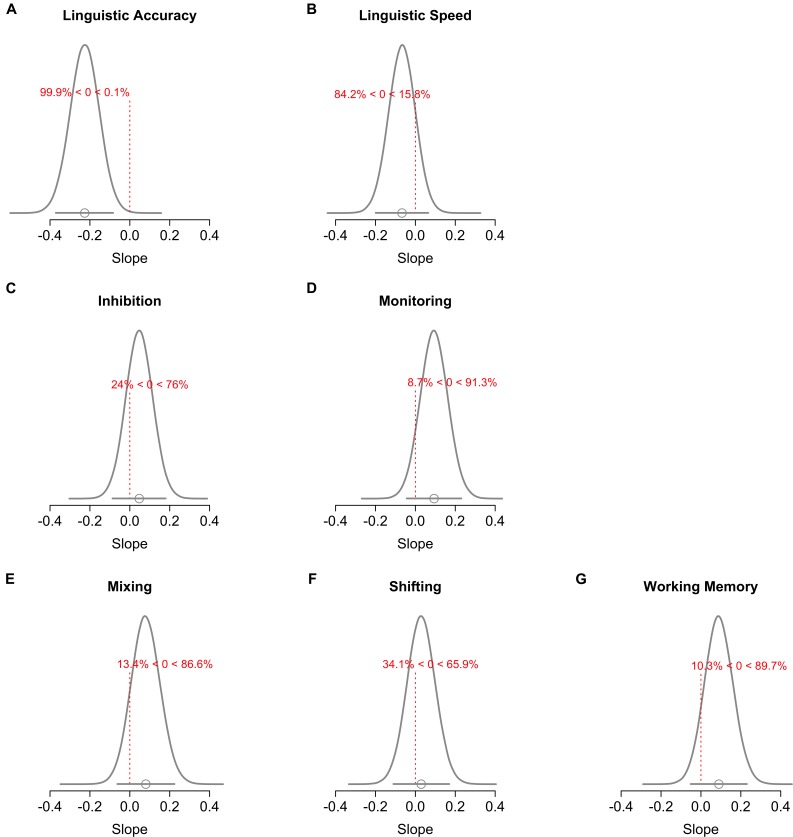
Posterior distribution of the slope of the language similarity effect for each cognitive ability. Each panel **(A–G)** shows the mean (dot) and the 95% HDI (bar underneath the curve) of the slope, and the proportion of the HDI that is below or above 0 (which represents the null).

Figure 3 presents the (*z*-transformed) measures as a function of language group and cognitive ability, and the predictions of the linear contrast over language similarity. The predictions represent the mean and the 95% highest-density interval (HDI) of the parameter posterior distribution. The HDI reflects the range of credible values of the parameter given the data. Figure 4 presents the posterior distribution of the slope of the linear contrast over language similarity for each cognitive ability. Panels in Figure 4 show the mean (circle underneath the curve) and the 95% HDI (bar underneath the curve) of the slope, and the proportion of the HDI that is above and below 0. Table 5 presents the BF_10_ for the effect of language similarity assessed by (a) the linear contrast over language group, (b) the sliding contrast comparing every two adjacent levels of the language similarity factor, as well as (c) the comparison of each group against the monolingual group (simple contrast).

**Table 5:** Evidence (BF_10_) for and against the effect of language similarity on each ability.

			Sliding contrast							Simple contrast		
				
Ability	Linear trend		Mono vs. Bidial		Bidial vs. Sim		Sim vs. Dissim		Mono vs. Sim		Mono vs. Dissim	

	**BF_10_**	**% error**	**BF_10_**	**% error**	**BF_10_**	**% error**	**BF_10_**	**% error**	**BF_10_**	**% error**	**BF_10_**	**% error**
Linguistic ability												
Linguistic accuracy	**30.20**	±1.46	**0.27**	±1.67	0.53	±2.30	1.64	± 0.72	**0.29**	±0.91	**7.11**	±0.84
Linguistic speed	0.45	±2.17	**0.29**	±2.20	**0.28**	±1.11	**0.27**	±1.82	0.39	±1.04	0.36	±**1.23**
Executive functions												
Inhibition	0.35	±1.41	**0.27**	±1.73	0.49	±0.96	0.34	±0.96	0.35	±1.44	**0.27**	±2.63
Monitoring	0.75	±0.91	1.07	±2.78	**0.26**	±1.88	**0.33**	±0.74	1.83	±1.10	0.43	±1.19
Mixing	0.68	±0.81	0.71	±3.97	0.45	±0.86	0.37	±1.37	1.11	±0.69	0.66	±0.91
Shifting	0.37	±0.98	**0.32**	±0.94	0.72	±1.56	1.02	±1.20	0.34	±1.24	**0.32**	±0.99
Working memory	0.74	±0.78	**3.85**	±1.83	0.39	±1.37	0.35	±1.05	1.24	±0.89	0.74	±0.75


*Mono, Monolinguals; Bidial, Bidialectals; Sim, Similar Bilinguals; Dissim, Dissimilar Bilinguals. All models included the average parents’ education level as a proxy for socio-economic status as an additional predictor. Values printed in bold indicate at least substantial evidence for (BF_*10*_ ≥ 3) or against (BF_*10*_ ≤ 0.33) the alternative hypothesis.*

### Linguistic Ability

Linguistic accuracy (see Figures 3A, 4A) and speed (Figures 3B, 4B) decreased as language similarity decreased. This trend was only credibly different from 0 for accuracy though (see Figure 4A), which was also reflected by the evidence being substantial for the presence of a linear effect of language similarity on accuracy but not speed (see Table 5). At the level of pairwise group comparisons, however, the pattern was more nuanced: Comparison of monolinguals against bidialectals and similar bilinguals showed substantial evidence for the null, whereas the comparison of monolinguals against dissimilar bilinguals yielded substantial evidence for a bilingual cost in linguistic accuracy. Other comparisons yielded ambiguous evidence for or against differences. In linguistic speed, pairwise comparisons of adjacent groups yielded substantial evidence against group differences, but the comparison of the monolingual group against the bilingual groups tended to show more ambiguous evidence against differences in this measure (see Table 5). As the word recognition accuracy score was unreliable (-0.13) and uncorrelated to the other linguistic-accuracy scores, we re-ran the analyses without this task. There was still substantial evidence for the linear effect of language similarity in accuracy (BF_10_ = 34.44 ± 0.74%). Removing SES from the linguistic processing analyses (see Table 6) did not change the pattern of results, except that the evidence against a linear effect on linguistic processing speed became substantial.

**Table 6:** Evidence (BF_10_) for and against the effect of language similarity on each ability (not controlled for SES).

			Sliding contrast						Simple contrast			
				
Ability	Linear trend		Mono vs. Bidial		Bidial vs. Sim		Sim vs. Dissim		Mono vs. Sim		Mono vs. Dissim	
						
	BF_10_	% error	BF_10_	% error	BF_10_	% error	BF_10_	% error	BF_10_	% error	BF_10_	% error
Linguistic ability												
Linguistic accuracy	**26.86**	±1.26	**0.26**	±0.88	0.53	±1.08	1.73	±0.58	**0.27**	±0.61	**10.41**	±0.45
Linguistic speed	**0.28**	±1.73	**0.25**	±1.10	**0.28**	±1.34	**0.26**	±1.38	0.36	±0.75	0.34	±0.92
Executive functions												
Inhibition	**0.25**	±1.00	**0.25**	±0.64	0.50	±0.53	**0.31**	±0.63	0.37	±3.02	**0.26**	±0.57
Monitoring	0.52	±0.78	0.80	±0.63	**0.30**	±2.01	**0.32**	±0.61	1.21	±0.66	0.59	±0.75
Mixing	0.47	±0.57	0.60	±0.61	0.44	±0.66	0.37	±0.88	1.57	±2.49	0.48	±1.76
Shifting	**0.25**	±0.61	**0.33**	±3.66	0.52	±2.26	0.90	±0.72	0.38	±1.75	0.38	±0.60
Working memory	0.59	±0.70	2.27	±0.88	0.38	±0.62	**0.32**	±1.16	1.35	±0.66	0.80	±2.93


*Mono, Monolinguals; Bidial, Bidialectals; Sim, Similar Bilinguals; Dissim, Dissimilar Bilinguals. Values printed in bold indicate at least substantial evidence for (BF_*10*_ ≥ 3) or against (BF_*10*_ ≤ 0.33) the alternative hypothesis.*

### EF Measures

Inspection of Figures 3C–G indicate a weak linear trend for better EF performance as language similarity decreases. Figures 4C–G show that the posterior distributions of the slopes tended to be positive, with 65.9% (Figure 4F) to 91.3% (Figure 4D) of the posterior values being larger than 0. However, Figures 4C–G also show that 0 was within the 95% HDI of all 6 slopes, hence indicating that a null effect is credible given the data. As shown in Table 5, although weak if not ambiguous, evidence was in favor of the null hypothesis over the alternative for a linear effect of language similarity for all EFs. In line with the linear trend analyses, the comparisons of adjacent levels of the language similarity factor showed mostly ambiguous evidence for or against group differences (see Table 5). There was substantial evidence against monolinguals and bidialectals performing differently in inhibition and shifting measures, but bidialectals outperformed monolinguals in WM performance. Evidence was largely ambiguous regarding the performance differences between bidialectals and similar bilinguals, except for monitoring, for which there was substantial evidence against group differences. The comparisons of similar and dissimilar bilinguals yielded mostly weak evidence for the null hypothesis, with exception of monitoring for which the evidence was substantial for the null over the alternative hypothesis. Lastly, when contrasting monolinguals against similar and dissimilar bilinguals, the evidence was mostly ambiguous. For inhibition and shifting, the evidence was even substantially supporting no group differences when comparing extreme groups (i.e., monolingual vs. dissimilar bilinguals).

Given the low correlation between the flanker and Simon inhibition scores and the low reliability of the flanker inhibition score, we also ran the analyses for each task separately (and for all other tasks as well; see Supplementary Table S1). For both tasks, the evidence remained overall ambiguous (BF_10_ between 0.53 and 1.88). We also ran all of the analyses reported here without entering SES (see Table 6). A similar pattern of results emerged, with the main difference being that inhibition and shifting yielded substantial evidence against an effect of language similarity (linear trend), and that the evidence for an effect of bidialectalism on WM was reduced to an ambiguous range (BF_10_ = 2.27 ± 0.88%). To rule out that SES drove the effect of bidialectalism on WM, we examined the main effect of SES and tested for an interaction between group (monolinguals vs. bidialectals) and SES: yielding evidence in the direction of the absence of both a main effect of SES (BF_10_ = 0.53 ± 1.46%) and of an SES x group interaction (BF_10_ = 0.40 ± 1.39%).

### Language Experience: Continuous Predictors

**FIGURE 5:**
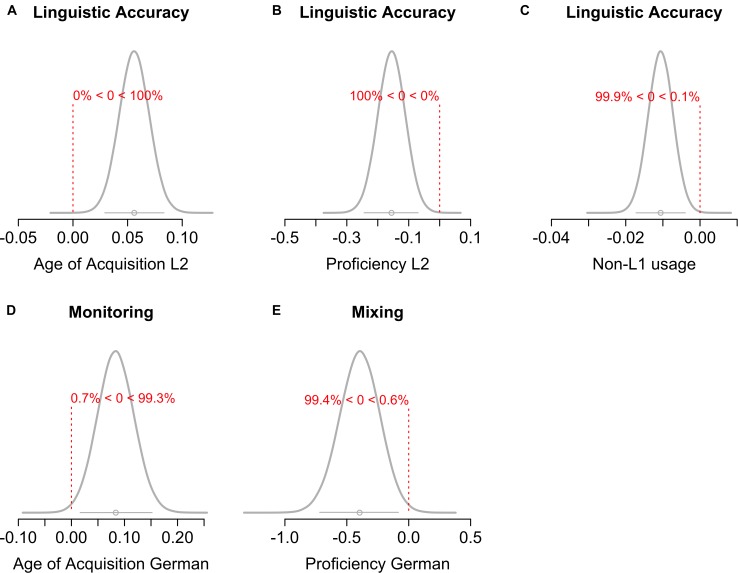
Posterior distribution of the effect of the continuous bilingual predictors that yielded substantial evidence for the alternative hypothesis over the null. Each panel **(A–E)** shows the mean (dot) and the 95% HDI (bar underneath the curve) of the effect, and the proportion of the HDI that is below or above 0 (which represents the null). Note that the x-axis varies between panels. Figures of the posteriors of the effects of all continuous predictors on all assessed abilities can be found on the OSF.

As the group design was mainly aimed to assess differences in linguistic ability and EF functioning due to language similarity, it might not have adequately captured effects of other aspects of the bilingual experience (e.g., the effect of age of aquiring a second language, the proficiency, or the frequency of using it in a daily context). Therefore, we additionally ran the analyses reported above using continuous measures of bilingualism as predictors instead of language group (see Figure 1 for all continuous predictors and their distribution in the language groups). We ran a separate model for each predictor to avoid issues with multicollinearity. All models included SES as covariate. Figure 5 shows the posterior distributions of the continuous predictors that yielded substantial effects. Figures with the posterior distributions of all continuous predictors for all abilities can be found on the OSF (Supplementary Figures S1–S3). The results largely reflected the findings reported for the group comparisons (see Supplementary Table S2). Substantial evidence for an effect of bilingualism emerged only for linguistic accuracy but not for linguistic speed. Specifically, a younger age of L2 acquisition (BF_10_ = 784.22 ± 1.43%) and higher L2 proficiency (BF_10_ = 104.47 ± 0.86%) was associated with lower linguistic accuracy (see Figures 5A,B). Moreover, a higher proportion of daily usage (BF_10_ = 34.83 ± 0.68%) of other languages besides German or the Swiss German dialect was associated with lower linguistic accuracy (Figure 5C). Notably, however, the effect was very small (*M* = -0.01). For EF, substantial evidence was only present for an effect of age of acquisition of German on monitoring (BF_10_ = 6.11 ± 1.23%; see Figure 5D) and German proficiency on mixing ability (BF_10_ = 8.24 ± 0.94%; see Figure 5E). As can be seen in Figures 5D,E, these effects were in the direction of a monolingual disadvantage, such that younger age of learning German and higher German proficiency was associated with lower EF performance.

## Discussion

Theoretical claims about the effects of bilingualism on EF rest on the assumption that the heightened demands for language control in bilinguals require general executive control processes, thereby providing lifelong EF training. Our main goal was to examine the putative link between the difficulty in managing two languages (reflected by costs in linguistic performance) and EF performance. For this purpose, we assessed several aspects of linguistic and EF processing in the same group of participants, and related their performance to their self-reported language experience. We classified participants in one of four groups with respects to language similarity (i.e., monolinguals, bidialectals, similar bilinguals, and dissimilar bilinguals) and tested for a linear effect of language similarity on linguistic and EF performance.

We predicted that language similarity should have opposite effects on linguistic and EF performance: language combinations that facilitate language control should yield comparatively better linguistic processing, but reduce the opportunities to train executive control, leading to limited EF advantages. We obtained evidence that language similarity was linearly related to linguistic accuracy, with similar languages yielding better performance than dissimilar languages. In line with the predictions, the estimated slope of the effect of language similarity on EF was indeed opposite to the slope of its effect on linguistic accuracy (i.e., better EF performance with more dissimilar languages). However, despite this trend, the evidence was overall ambiguous and tended to support the null hypothesis, with one exception: we found substantial evidence for a positive association between bidialectism and WM.

### Language Similarity, Linguistic Ability, and EF

The observed effect of language similarity on linguistic processing is consistent with several other reports in the literature (but see Costa et al., 2006). Previous studies have found that bilingual children with more similar languages (e.g., Spanish-English) outperform bilingual peers with less similar languages (e.g., Chinese-English) in linguistic tasks (Bialystok et al., 2003, 2005; Barac and Bialystok, 2012). This pattern also extends to young adults: in a sentence production task, dissimilar bilinguals (Mandarin-English) showed larger bilingual disadvantages, as measured by higher frequency effects, than similar bilinguals (Spanish-English; Runnqvist et al., 2013). Taken together with our results, these findings support the notion that linguistic adaptation is more pronounced for more similar languages. Furthermore, our results indicate that L2 can have an impact on L1, corroborating recent studies showing bidirectional cross-linguistic effects (e.g., Hohenstein et al., 2006; Brown and Gullberg, 2008, 2010, 2011; Ameel et al., 2009). This is in line with the assumption that both languages are activated simultaneously in the bilingual mind, leading to cross-interactions between languages. Our findings indicate that these adaptations facilitate language processing when languages are similar, thereby arguably reducing executive control demands. Hence, bilinguals speaking more dissimilar languages (which face the most challenging linguistic condition) should show larger EF benefits. However, the evidence was ambiguous (BF_10_ between 0.35 and 0.75 for a linear trend) for effects of language similarity on all of the EFs assessed.

Besides language similarity, the bilingual linguistic disadvantage found here might also reflect an effect of language usage (i.e., bilinguals generally use each of their languages less often than monolinguals use their L1). Support for this hypothesis comes from studies showing that lexical access in both L1 and L2 is delayed in bilinguals relative to monolinguals, specifically for less frequently used words (e.g., Gollan et al., 2008; Ivanova and Costa, 2008). However, in the present study, some monolinguals also indicated to use languages other than their L1 on a frequent basis. Furthermore, in a follow-up analysis examining the effect of continuous variables of the bilingual experience, we found that besides higher frequency of using non-L1 languages, higher proficiency and lower age of acquisition in L2 were also associated with lower linguistic accuracy across groups. Thus, taken together, frequency of language usage alone cannot entirely explain the group differences reported here.

### Bidialectalism and Its Association With WM

Considering bidialectalism as an extreme case of language similarity is one novel feature of the present study. Recent studies in the field have suggested that bidialectalism may involve similar language control demands as bilingualism (Kirk et al., 2014; Antoniou et al., 2016) and may, thus, yield similar EF benefits. To our best knowledge, our study is the first to test how bidialectism affects both linguistic abilities and EFs in a sample of young adults. Our results showed evidence that bidialectism was associated with better WM performance than monolingualism. However, we did not observe additive effects of speaking an additional language (i.e., bidialectals did not differ from bilinguals), and the WM benefits did not generalize to any other of the assessed EFs.

One may argue that the presence of this effect solely for WM is in line with recent notions that, given the central role of executive control in WM (Baddeley, 1986; Cowan, 1995; Engle, 2002; Oberauer and Hein, 2012), any effects of speaking an L2 can be expected to be stronger for WM tasks than for any other EF tasks (Bialystok, 2017). Bidialectal effects on executive control may, hence, simply be too subtle to be detected in non-WM tasks with less executive control demands. However, this explanation is contrary to findings from Miyake and Friedman (2012; see also Friedman et al., 2008) showing that the contribution of WM performance to a general executive control factor (i.e., “common EF”) is not particularly greater than the contribution made by shifting or inhibition performance to that factor.

Therefore, taken together with the effect on WM not being modulated by language similarity, the absence of an effect on other EFs could suggest that bidialectism does not practice common executive control as much as it does processes that are more specific to WM – for example the access and retrieval of currently relevant information. However, the present data do not allow for directly testing this proposition. Future studies specifically designed to disentangle effects of bidialectism and bilingualism on executive control from effects on retrieval of information may shed further light on the specific cognitive mechanisms affected by bilingualism.

### Limitations

Although we found substantial evidence for a linear trend of language similarity on linguistic performance, evidence was mostly weak for the pairwise comparisons of adjacent levels of this factor. This is likely due to the fact that performance differences between two adjacent levels were too small to be distinguished from within-group variability by data from only about 24 participants per group. Therefore, future studies aiming at examining the effects of contrasting only two levels of language similarity on linguistic performance may need substantially larger group sizes.

Similarly, many of the effects on EF (linear trend and group contrasts) yielded evidence within the ambiguous range (i.e., BF_10_ between 0.34 and 1.83). The ambiguous results obtained here are in line with recent concerns that studies with small sample sizes provide ambiguous and unreliable evidence for effects of bilingualism on EF performance. Even with a total sample of 99 students and using a linear contrast approach, which is more powerful to detect experimental effects, we were unable to firmly reject or support the hypothesis that language similarity influences EF performance. In fact, for any EF considered here, the absolute slope of the language similarity effect was substantially smaller than that for linguistic accuracy, which indicate that the effects, if they were true, would be harder to detect with the small sample sizes common in the bilingual advantage literature.

Furthermore, we observed substantial effects of language similarity on linguistic accuracy but not speed. As effects in accuracy and speed can show trade-offs (Wickelgren, 1977), future studies may consider using sequential sampling models (such as the diffusion model; Ratcliff, 1978), which integrate information across these two measures to derive psychologically meaningful parameters. This may be a fruitful venue to examine how language experience affects different cognitive processes involved in decision-making.

Regarding the inclusion criteria of the present study, we chose age of acquisition (cutoff 7 years) and continous active language usage as requirements for categorizing participants as bilingual, with the aim to most closely align our definition with previous studies testing for bilingual EF advantages (Bialystok et al., 2006; Costa et al., 2009; Hernández et al., 2010; Prior and MacWhinney, 2010; Luk et al., 2011; Gold et al., 2013). However, some studies have also found advantages in EF for bilinguals with a later age of acquisition (Pelham and Abrams, 2014; Vega-Mendoza et al., 2015). Thus, it is possible that factors of language experience other than the early acquisition of a language might bear more explanatory value for the proposed training effect on EF functioning (see below for a more detailed discussion of this topic).

Even though we paid close attention to match the groups and included random effects to take individual differences between participants and also between tasks of a measured ability into account (Coderre and van Heuven, 2014), we still faced the problem of group differences that were unrelated to bilingual status. These differences primarily affected the monolinguals. First, monolinguals differed from bidialectals and bilinguals regarding their immigrational background. Second, monolinguals in the present study may be considered as less monolingual than those in other studies reporting a bilingual advantage in EF (e.g., Luo et al., 2013) as they had, on average, learned at least three languages at a later point in their lives. Specifically, two monolinguals indicated to use a non-native language around half of the time or more. Additional analyses using continuous predictors of bilingualism revealed that higher non-L1 language usage was associated with lower linguistic accuracy. Therefore, if anything, excluding these individuals from the monolingual group would have resulted in an even stronger linguistic advantage of monolinguals over bidialectals and bilinguals. Although a monolingual group with less language exposure may have been desirable for the present study, this matches the Swiss (and European) demographic: learning two foreign languages is required by the Swiss educational system, and people in Switzerland speak on average two languages besides their native language (Werlen, 2009). Thus, we cannot rule out that a comparison to a strictly monolingual and non-immigrant sample would have led to stronger effects of bilingualism. However, in an increasingly globalized world the number of people speaking more than one language is rising, and monolinguals not exposed to other languages at all are rare (Grosjean, 2010). For example, estimates from survey data suggest that more than half of the European population are able to hold a conversation in at least one additional language besides their L1 (European Commission, 2012), and approximately 21% of the U. S. American population speak a language other than English at home (US Census Bureau, 2015). If dialects were also counted as separate languages, these percentages would rise even higher. Hence, effects for individuals without any L2 exposure at any time in their lives are not informative for the majority of the population. Moreover, as discussed previously (von Bastian et al., 2016), monolinguals without any L2 exposure will likely differ from bilinguals in other aspects than just language exposure that would then be confounded with any effects of bilingualism. Thus, any differences observed for such extreme groups may disappear when accounting for the full range of individuals (e.g., see Unsworth et al., 2015).

Lastly, as the present study was a first attempt to investigate the effect of language similarity on the link between linguistic and EF performance, we chose to define the similarity of two languages based on their common ancestry (i.e., language family), a perhaps overly simplified classification. This definition resulted in a large heterogeneity in our group of participants with regards to the exact language combinations they had acquired. Future research using a more fine-grained operationalization of language similarity is required to identify the specific language properties (e.g., lexical, phonological, or grammatical overlap) underlying the effects on linguistic accuracy found in the present study. Deriving quantitative predictions based on the precise degree of overlap between languages would allow for testing specific hypotheses regarding which aspects of language overlap are relevant in yielding cross-linguistic interactions and potential knock-on effects on EF advantages.

### Bilingualism Advantages: Challenges and Opportunities

As mentioned above, one limitation of this study is that we classified individuals as monolinguals or bilinguals based only on their age of acquisition and active usage of their L2. The choice of these criteria was based on previous literature at the time of designing this study. Arguably, however, it is possible that (an)other language background variable(s) would have been more predictive for linguistic and EF performance. For example, Figure 1 illustrates that all participants learned some other language(s) at some point later in their life (panel C), and most participants, including some monolinguals, used other languages on a daily basis (panel E). Furthermore, considerable heterogeneity existed within language groups with regards to these factors. Thus, depending on the definition one has of bilingualism, the participants in the present study could be regrouped in different ways. This is one of the limitations of groups created based on observed variables (i.e., quasi-experimental designs).

Although typically not in the focus of bilingual advantage research, it is possible that, in previous studies relative to the present study, the number of languages learned and/or the non-L1 language usage (or any other potential indicator of bilingualism) were more closely aligned with age of acquisition and active usage – and, so, possibly the actual driving forces behind the bilingual advantages found. Indeed, assuming bilingual EF advantages exist in the first place, it is not unlikely that no single language background variable can explain bilingual advantages in their entirety but that (only) a certain combination of language experiences leads to EF advantages. However, it is yet unclear what combination of variables might be important to describe bilingualism, let alone what instruments are best suited to measure bilingualism (Surrain and Luk, 2017). Moreover, bilingualism may not be a static trait but evolve dynamically over time depending on many other circumstances including neccessity, context, and social and societal norms of language usage. One challenge is, therefore,to better capture the multidimensional and dynamic reality of bilingualism. To some extent, this development is already happening, with most studies using a multi-method approach combining self-reported measure (e.g., the Language Experience and Proficiency Questionnaire, LEAP-Q, Marian et al., 2007) with performance-based assessments (e.g., the Multilingual Naming Test, MINT, available in multiple languages, Gollan et al., 2012). Moreover, recent studies have adopted a multidimensional understanding of bilingualism (Luk and Bialystok, 2013) by using a range of different variables, such as proficiency and age of L2 acquisition, as continuous predictors (e.g., Paap et al., 2014; von Bastian et al., 2016). Similarly, in the present study, we ran a series of additional analyses using continuous indicators of bilingualism as predictors of linguistic and EF functioning, resulting in largely the same pattern of results as for the group design. Without clear theoretical predictions as to which language background variables (or combinations thereof) should relate to bilingual EF advantages, however, the selection and reporting of those variables will remain relatively unsystematic and, so, studies will be at risk of turning into analytical fishing expeditions.

Over and above the challenges of selecting, assessing, and analyzing dimensions of bilingualism, relatively vague theorizing poses an additional challenge. To meet this challenge in the present study, we tested the broad notion of bilingual EF advantages by examining one specific, theoretically derived, mechanism – the similarity of the two languages spoken – as a proxy of the demands of cross-language interference and its effect on both linguistic and EF performance. We did not find evidence to support the predicted relationship and, thus, our findings question the theoretical validity of the cross-language interference serving as the link between language experience and EF advantages.

As any single study, our findings are not definitive and require replication, and possibly theoretical and methodological refinement. In bilingual advantage research, replication poses a particular challenge due to the many sources of variation in measurement and sampling. Open Science practices, as followed in the present study, can support both theory development and replication attempts (e.g., Munafò et al., 2017). First, by providing our dataset alongside the analysis scripts, other researchers can directly test alternative analytical procedures and alternative hypotheses using our data. For example, the participants in this sample could be regrouped according to a different definition of bilingualism or of language similarity. Second, by providing open materials that can be used with open-source experimental software such as Tatool Web, other researchers can attempt an exact replication of our study with larger sample sizes or with a refined definition of language similarity. Third, pooling the present data set with data from such replication attempts will lead to more precise estimates of the effects of language experience on linguistic and non-linguistic tasks.

## Conclusion

We found that the similarity of the two languages spoken by bidialectals and bilinguals affects linguistic processing in a linear fashion, with performance worsening the more dissimilar the two languages are. However, the increased difficulty of managing two more dissimilar languages did not translate into substantial evidence for increased EF benefits. We contend that any fruitful future investigation in the field needs to test clear theoretical links between language demands and EFs, as advanced here.

## Ethics Statement

This study was carried out in accordance with the recommendations of the ‘Ethical Principles of Psychologists and Code of Conduct” of the American Psychological Association (APA) and the ‘Ethische Richtlinien für Psychologinnen und Psychologen der Schweizerischen Gesellschaft für Psychologie (SGP) of the SGP with written informed consent from all subjects. All subjects gave written informed consent in accordance with the Declaration of Helsinki. The protocol was approved following the self-assessment checklist for ethical treatment of human participants provided by the Ethics Committee of the Faculty of Arts and Social Sciences of the University of Zurich.

## Author Contributions

The research reported in this paper was derived from two master theses submitted to the University of Zurich by JO under the supervision of ASS and from AS under the supervision of CvB. All authors contributed to the conception and design of the study. JO and AS collected the data. JO and ASS performed the statistical analysis. JO wrote the first draft of the manuscript. ASS and CvB wrote sections of the manuscript. All authors approved the submitted version.

## Conflict of Interest Statement

The authors declare that the research was conducted in the absence of any commercial or financial relationships that could be construed as a potential conflict of interest.

## Footnotes

1 This classification is at a broad ordinary level, as we had no means of computing the degree of language overlap between all language combinations reported by our participants.

2 One participant was native speaker of a dialect of German other than Swiss German (i.e., Austrian), and hence this person could not be classified into either the monolingual or bidialectal groups. The other six participants qualified as similar bilinguals but used a different (non-Latin) script in one of their languages. We excluded these participants to keep script similarity constant in the similar bilingual group.

3 Before and after this task, participants also completed single-list blocks which were similar with the exception that only one list was memorized and consequently there was no switching between lists. In the present study, we considered only performance in the two-list block, because this condition places a higher demand on WM capacity which has been suggested as important for observing a bilingual effect (see Bialystok et al., 2004).

## Supplementary Material

FIGURE S1 Posterior distribution of the effect of age of acquisition (AoA) of German, of the Swiss German dialect, and of the second language (L2). Each panel **(A–G)** shows these effects for each cognitive ability. The dot and the bar underneath the curve shows the mean and the 95% HDI of the posterior, respectively.

FIGURE S2 Posterior distribution of the effect of proficiency (Prof.) in German, in the Swiss German dialect, and in the second language (L2). Each panel **(A–G)** shows the posterior of these effects for each cognitive ability. The dot and the bar underneath the curve shows the mean and the 95% highest density interval (HDI) of the posterior, respectively.

FIGURE S3 Posterior distribution of the effect of the number of languages learned (N. Lang.), the number of languages learned below age 7 (N. Lang. < 7), and the % of language usage other than the first language (Non-L1 Usage). Each panel **(A–G)** presents the posterior of these effects for each cognitive ability. The dot and the bar underneath the curve shows the mean (dot) and the 95% HDI of the posterior, respectively.

TABLE S1 Evidence (BF_10_) for (and against) the effect of language similarity on each task. Values printed in bold indicate at least substantial evidence for (BF_10_ = 3), and values printed in gray against (BF_10_ = 0.33) the alternative hypothesis.

TABLE S2 Evidence (BF_10_) for (and against) a linear effect of the continuous language demographic variables on each ability. Values printed in bold indicate at least substantial evidence for (BF_10_ = 3), and values printed in gray against (BF_10_ = 0.33) the alternative hypothesis.
