# Supplementary figures and images for: Bidialectalism and Bilingualism: Exploring the Role of Language Similarity as a Link Between Linguistic Ability and Executive Control
Source: Front Psychol. 2018 Oct 23;9:1997. doi: 10.3389/fpsyg.2018.01997 (PMC6206588; doi:10.3389/fpsyg.2018.01997)

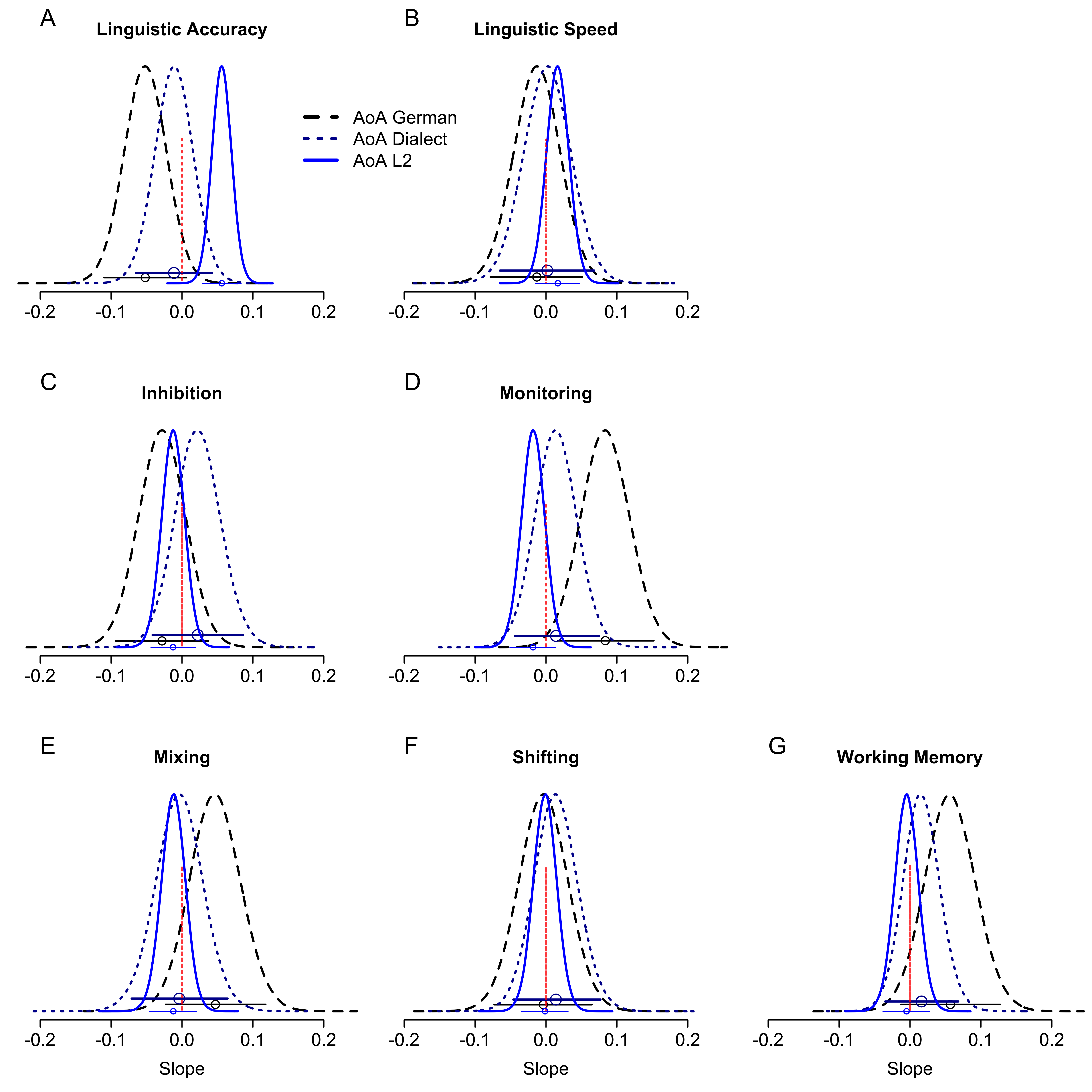

Supplement: FIGURE S1 — Posterior distribution of the effect of age of acquisition (AoA) of German, of the Swiss German dialect, and of the second language (L2). Each panel (A–G) shows these effects for each cognitive ability. The dot and the bar underneath the curve shows the mean and the 95% HDI of the posterior, respectively. [file Image_1.tiff]

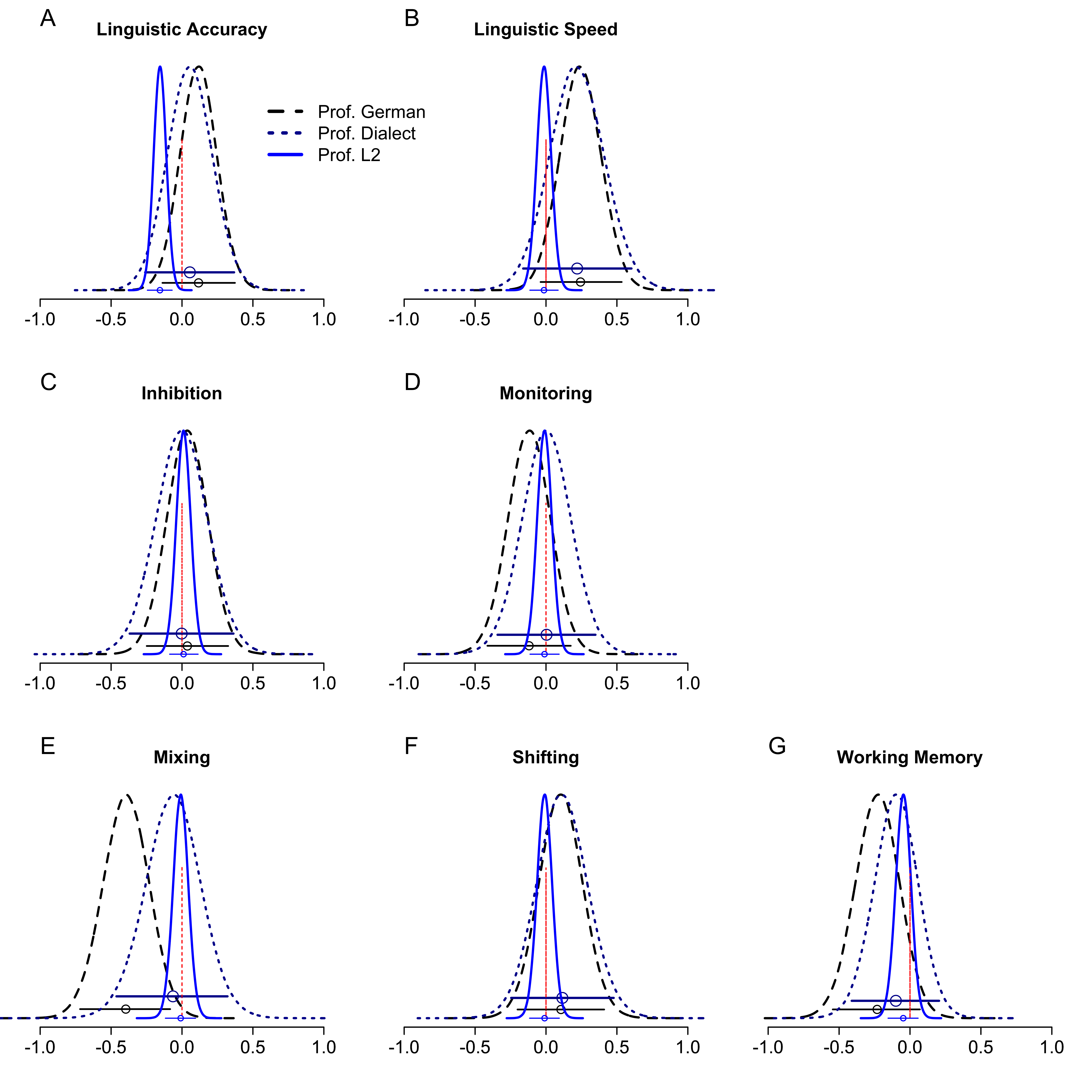

Supplement: FIGURE S2 — Posterior distribution of the effect of proficiency (Prof.) in German, in the Swiss German dialect, and in the second language (L2). Each panel (A–G) shows the posterior of these effects for each cognitive ability. The dot and the bar underneath the curve shows the mean and the 95% highest density interval (HDI) of the posterior, respectively. [file Image_2.tiff]

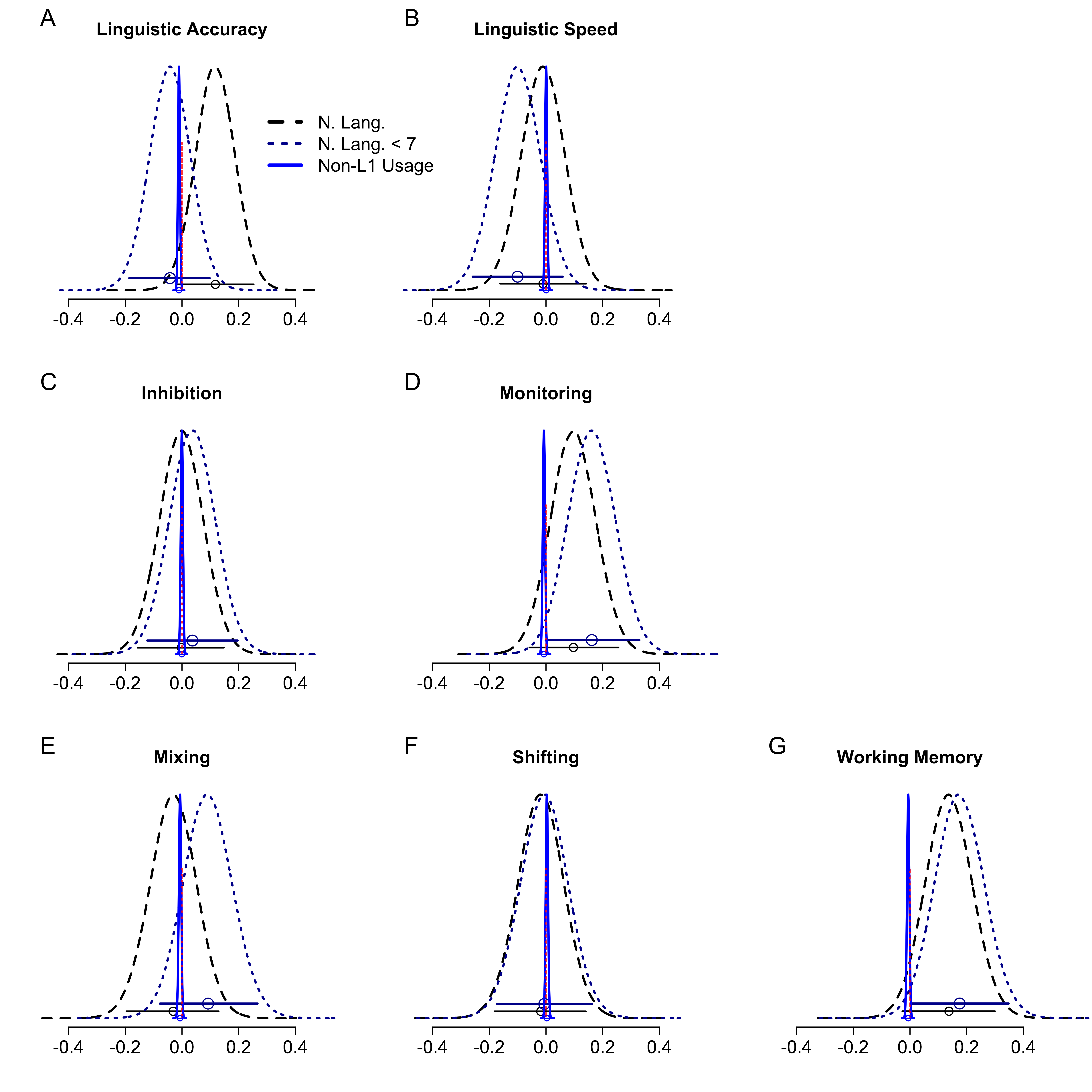

Supplement: FIGURE S3 — Posterior distribution of the effect of the number of languages learned (N. Lang.), the number of languages learned below age 7 (N. Lang. < 7), and the % of language usage other than the first language (Non-L1 Usage). Each panel (A–G) presents the posterior of these effects for each cognitive ability. The dot and the bar underneath the curve shows the mean (dot) and the 95% HDI of the posterior, respectively. [file Image_3.tiff]
